# Rosinidin Protects against Cisplatin-Induced Nephrotoxicity via Subsiding Proinflammatory and Oxidative Stress Biomarkers in Rats

**DOI:** 10.3390/ijerph19159719

**Published:** 2022-08-07

**Authors:** Sadaf Jamal Gilani, May Nasser Bin-Jumah, Fahad A. Al-Abbasi, Muhammad Shahid Nadeem, Sami I. Alzarea, Mohammed Muqtader Ahmed, Nadeem Sayyed, Imran Kazmi

**Affiliations:** 1 Department of Basic Health Sciences, Preparatory Year, Princess Nourah bint Abdulrahman University, Riyadh 11671, Saudi Arabia; 2 Biology Department, College of Science, Princess Nourah bint Abdulrahman University, Riyadh 11671, Saudi Arabia; 3 Environment and Biomaterial Unit, Health Sciences Research Center, Princess Nourah bint Abdulrahman University, Riyadh 11671, Saudi Arabia; 4 Saudi Society for Applied Science, Princess Nourah bint Abdulrahman University, Riyadh 11671, Saudi Arabia; 5 Department of Biochemistry, Faculty of Science, King Abdulaziz University, Jeddah 21589, Saudi Arabia; 6 Department of Pharmacology, College of Pharmacy, Jouf University, Sakaka 72341, Saudi Arabia; 7 Department of Pharmaceutics, College of Pharmacy, Prince Sattam bin Abdulaziz University, Al-Kharj 11942, Saudi Arabia; 8 Glocal School of Pharmacy, Glocal University, Saharanpur 247121, India

**Keywords:** nephrotoxicity, cisplatin, rosinidin, oxidative stress, catalase, glutathione

## Abstract

Background: Rosinidin is a flavonoid anthocyanin pigmentation found in shrub flowers such as *Catharanthus roseus* and *Primula rosea*. The molecular docking studies predicted that rosinidin has adequate structural competency, making it a viable medicinal candidate for the treatment of a wide range of disorders. The current study intends to assess rosinidin nephroprotective efficacy against nephrotoxicity induced by cisplatin in rats. Materials and Methods: Oral acute toxicity tests of rosinidin were conducted to assess potential toxicity in animals, and it was shown to be safe. The nephroprotective effect of rosinidin 10, and 20 mg/kg were tested in rats for 25 days with concurrent administration of cisplatin. Several biochemical parameters were measured to support enzymatic and non-enzymatic oxidative stress such as superoxide dismutase (SOD), malondialdehyde (MDA), and glutathione peroxidase (GSH). Likewise, changes in several non-protein-nitrogenous components and blood chemistry parameters were made to support the theory linked with the pathogenesis of chemical-induced nephrotoxicity. Results: Cisplatin caused significant changes in biochemical, enzymatic, and blood chemistry, which rosinidin efficiently controlled. Conclusions: The present investigation linked rosinidin with nephroprotective efficacy in experimental models.

## 1. Introduction

Chronic kidney disease (CKD) is one of the most serious public health issues in the world, affecting a large proportion worldwide (8–16%) [1]. The main clinical features of CKD are the progressive loss of essential functions leading eventually to chronic kidney failure, which necessitates dialysis or kidney transplants to maintain quality of life [2]. Furthermore, earlier research has shown that acute kidney disease (AKD) connected with high prevalence of CKD, and individuals with CKD exacerbated by AKD have a higher mortality rate [3]. With the constantly rising prevalence of AKD in recent years, the death rate has risen dramatically [4]. Previous research found that several variables, including drugs, illnesses, eating habits, ischemia, and infection, showed their role in the pathology of AKD [5,6]. Previous data also suggests that drug-induced nephrotoxicity, in particular, is a critical contributor in around 60% of AKD cases in hospital settings [7].

The term nephrotoxicity refers to a condition in which toxic agents and drugs reduce the excretion of toxic metabolic products in the kidneys. There is considerable evidence that about 20% of nephrotoxicity is caused or induced by drugs [8]. As life expectancies increase and people take more medications, the percentage increases. Moreover, the previous findings postulated that chemotherapeutic agents and anticancer medications are undergoing major issues as they have the potential for nephrotoxicity and hence their implications in the treatment are possibly restricted [9].

Earlier findings established that around 20% of adults in the United States are affected by drug-induced nephrotoxicity, causing an annual impact of over 1.5 million adverse events [9,10]. 

However, findings suggested that the treatment cost for kidney ailment is estimated at several billion, even though most kidney damage is reversible [11]. Multiple pathways contribute to drug-induced nephrotoxicity, comprising renal tubular cytotoxicity, altered glomerular hemodynamics, and inflammation [12,13,14]. Researchers have conducted extensive studies on direct nephron-toxic mechanisms in the renal proximal tubule epithelial cells. There are, however, many transporters expressed by renal tubular epithelial cells, many of which are unique to each segment of the renal tubule. The result is the death of specific nephron fractions when drugs with an affinity for these transporters are administered [15,16,17]. 

Conversely, some medications which are reported as renal tubular toxins are reported to cause severe kidney injury by damaging the tubular epithelium-lining tissues. Moreover, studies also show that drug penetration can damage the epithelial cells in renal tubules, as a result of drug-associated ischemic episodes or urolithiasis. Previous data also suggested that nephropathies were associated with the several diagnostic agents used in the radiographic process such as contrast agents [18]. Another piece of evidence also explored the nephrotoxic events associated with several drugs that lead to scarring and inflammation of the renal tubular epithelium, which is known to cause renal fibrosis [19]. Several investigations have shown that glomerulonephritis is an inflammatory kidney condition induced by a variety of medications and infectious pathogens that is related to proteinuria [20]. 

The pieces of evidence of drug-induced toxicity are extensive; diagnostic and therapeutic data suggest that cisplatin (CP) causes oxidative stress in the kidneys, which leads to tubule destruction [21,22,23,24]. Earlier research found that reactive species of both oxygen and nitrogen (ROS, RNS) alter membrane functional and structural integrity during mitochondrial respiration [25,26]. Furthermore, the mechanisms for CP-induced acute nephropathy were explained through the accumulation of these proteins in kidneys and lysosomes [26,27]. CP-induced nephropathy can be caused by a variety of processes, such as inflammation, oxidative stress, apoptosis, and mitochondrial dysregulation. Nevertheless, the mechanism behind the malfunctioning is not completely known [28].

Evidence suggests that anthocyanin and its sugar-free analog, anthocyanidin, are red–blue water-soluble flavonoids present in higher plants, flowers, and fruits. Various foods and pharmaceutical ingredients utilize anthocyanin and anthocyanidin as colorants [29]). Additionally, various studies are being conducted to evaluate the health implications of anthocyanin and anthocyanidin. Researchers examined rosinidin, a flavonoid anthocyanins color found in the flowers of shrubs such as *Catharanthus roseus* and *Primula rosea*. Rosinidin stereochemistry revealed that it is composed of benzopyrylium, which includes hydroxyl substituents at ring positions three and five, followed by methoxy substituents at position seven, and a 4-hydroxy-3-methoxyphenyl substitution at position two [29]. 

The molecular docking investigations hypothesized that rosinidin has acceptable conformational competence, which could make it a suitable therapeutic contender for the treatment of various illnesses [30]. An additional line of research suggested that rosinidin restored the oxidative stress parameters in several diseases, and could be utilized as a beneficial compound in oxidative stress-mediated pathophysiology [30].

## 2. Materials and Methods

### 2.1. Animals

Male Wistar rats measuring 200–250 g was kept in conventional laboratory circumstances with (*n* = 6 per group) 12/12 h light/dark cycle components, 24 ± 3 °C temperature, and 40–50% humidity levels. A normal diet and water were offered to the animals before the experiment. It was established that rats should be acclimated over seven days following standard laboratory protocols. Institutional Animal Ethics Committee (IAEC/TRS/PT/021/0010), Ministry of Environment and Forests, Government of India, New Delhi gave final approval for the rodent experiments.

### 2.2. Drugs and Chemicals

For the present study, Sigma Chemical Co. (St. Louis, MO, USA) provided cisplatin. Rosinidin (isolated compound from *Catharanthus roseus*) was obtained gift sample from SRL, India. In addition, other chemicals used in the present investigation were of analytical grade from authentic source. 

### 2.3. Acute Toxicity Studies

In the present investigation, OECD ANNEX-423 standards were used to assess rosinidin acute oral toxicity (LD50). As per the previous toxicity studies, rosinidin was administered to rats orally and diluted in a sodium CMC solution (0.5 percent *w*/*v*) [31].

### 2.4. Investigational Plan

A slight modification to previous studies has been made for this proposed investigation [32]. There were 4 groups of 6 rats each, divided randomly. Both the normal (normal) and the cisplatin (cisplatin) groups (Group I and II) received daily vehicle treatment for 25 days. Animals were in Group III, and IV received rosinidin intraperitoneally at a dose of 10, and 20 mg/kg, respectively. The rats were given 4 injections of cisplatin (3 mg/kg body weight/day) in saline solution (0.9%) every five days for 25 days to be subject to nephrotoxicity in rodents. We determined body weights, and then the animals were euthanized under light ether anesthesia on the last day (25 days) of the study [33,34].

### 2.5. Biochemical Estimation

#### 2.5.1. Kidney Tissue Homogenate and Biological Sample Preparations 

The 25th day was employed for the collection of urine samples from 24-h urine via metabolic cages. Following light anesthesia, blood was composed and subjected to centrifugation at 1000 r.p.m for 20 min to separate the serum. A urine and serum sample was cast-off to evaluate the different biochemical parameters. The kidneys of the rats were removed and cleaned with regular saline solution after they was sacrificed. To determine several biochemical parameters including superoxide dismutase (SOD), malondialdehyde (MDA), and glutathione peroxidase (GSH), 10% *w*/*v* tissue homogenates were organized with 0.1 M Tris–HCl buffer (pH 7.5) followed by centrifugation at 3000 r.p.m for 15 min [28,32]. 

#### 2.5.2. Assessment of Urea, Uric Acid, Creatinine, and Creatinine Clearance

Employing widely viable diagnostic kits, serum (254 nm) and urine (340 nm) were spectrophotometrically examined (Hitachi’s Double-Beam UV/Vis Spectrophotometer-U-2000, Hitachi High-Tech, Haryana, India) to assess urea, uric acid, and creatinine levels. Creatinine clearance is a marker for glomerular filtration rate that can be calculated by a well-established methodology and formula.

Creatinine clearance (mL/min):

$$\frac{\left[\mathrm{Urinary}\;\mathrm{Cr}\;\left(\mathrm{mg}/\mathrm{dL}\right)\times 24\;h\;\mathrm{urine}\;\mathrm{volume}\;\left(\mathrm{mL}\right)\right]}{[\mathrm{Serum}\;\mathrm{Cr}\;(\mathrm{mg}/\mathrm{dL})\;\times 24\times 60\;\min]}$$

#### 2.5.3. Assessment of Blood Urea Nitrogen (BUN), Phospholipid, and Cholesterol

A conventional technique was used to determine the phospholipid content in serum [35]. The determination of serum cholesterol and blood urea nitrogen (BUN) levels was performed using commonly available assays methods (Agapee Diagnostics, Maharashtra, India).

#### 2.5.4. Estimation of Biomarkers of Oxidative Stress

The essays were determined using the method of previous researcher serum GSH [36], SOD [37], catalase activity (CAT) [38], MDA [39] and GSSG reductase (GR) [40] using commercial assay kits from Transasia Biomedical Limited, Mumbai, India. 

### 2.6. Statistical Analysis

For the present investigation statistical analysis was carried out by employing Prism software version 5.02 (Graph Pad Software, San Diego, CA, USA). In this statistical analysis, results were abridged as mean ± SEM. Similarly, Kruskal–Wallis test has been used for data analysis, and the values at *p* < 0.05 will consider as statically significant.

## 3. Results

### 3.1. Evaluation of Acute Toxicity

Acute oral toxicity investigations in the current study demonstrated that rats given a rosinidin with maximum dosage orally were found to be safe. Through 14 days, no clinically evident symptoms or morbidity were observed. Based on findings from acute oral toxicity experiments, 10 mg/kg and 20 mg/kg, were chosen for further investigation.

### 3.2. Estimation of Non-Protein-Nitrogenous Components

#### Effect of Rosinidin on the Estimation of Non-Protein-Nitrogenous Components against Cisplatin-Induced Nephrotoxicity in Rats

**Figure 1:**
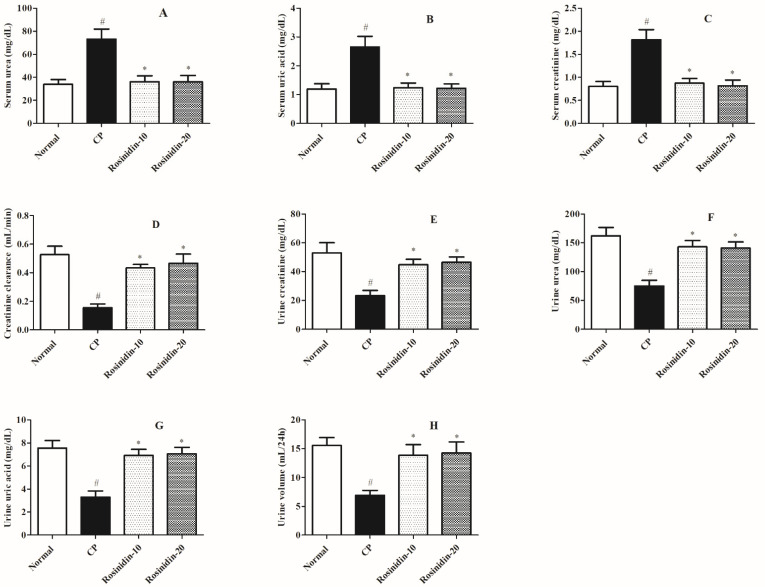
Effect of rosinidin on the estimation of non-protein-nitrogenous components against cisplatin-induced nephrotoxicity in rats. (**A**) Serum Urea (**B**) Serum Uric Acid (**C**) Serum Creatinine (**D**) Creatinine Clearance (**E**) Urine Creatinine (**F**) Urine Urea (**G**) Urine Uric Acid (**H**) Urine Volume. Data were presented as mean ± SEM (*n* = 6). # Indicate significant alteration for normal vs. control, * indicated modulations observed in rosinidin vs. cisplatin. *p* value less than 0.05 were considered statistically significant. The analysis was carried out by employing Kruskal–Wallis test.

Figure 1 illustrates the influence of rosinidin on the account of non-protein-nitrogenous components in rats with cisplatin-induced nephrotoxicity. During the current study, the cisplatin control group had substantially higher levels of serum urea, uric acid, and creatinine than the usual treatment group (*p* < 0.05), which is indicative that there is a significant alteration in the excretion of the above-mentioned parameters in the cisplatin-induced groups. Similarly, the current investigation concurrently analyses the urine parameters to portray urine parameters such as urine volume, urea, creatinine, uric acid, and creatinine clearance to demonstrate the hazardous levels of the above-mentioned elements as a consequence of cisplatin-induced nephrotoxicity in rats. Hence, in another set of analyses, when compared to the normal-treated group the cisplatin-induced rats showed a significant decrease in urine volume, urine urea, urine creatinine, urine uric acid, and creatinine clearance (*p* < 0.05). Furthermore, a Kruskal–-Wallis test revealed that treatment with rosinidin (20 mg/kg) significantly restored the elevated levels of serum urea (*p* < 0.01), uric acid (*p* < 0.05), and creatinine (*p* < 0.05). Similarly, remarkable alterations were observed in the urine parameters such as urine volume (*p* < 0.05), urine urea (*p* < 0.05), urine creatinine (*p* < 0.05), urine uric acid (*p* < 0.05), and creatinine clearance (*p* < 0.05) in rats treated with a high dose of rosinidin (20 mg/kg) as compared with cisplatin-treated rats. However, rats treated with a low dose of rosinidin (10 mg/kg) showed less significant modulation in the serum profiles such as serum urea, uric acid, and creatinine (*p* < 0.05) as compared to cisplatin-treated rats. Similarly, while evaluating urine parameters, low-dose rosinidin (10 mg/kg) exhibits poor modulations in the decreased levels of urine volume, urine urea, urine creatinine, urine uric acid, and creatinine clearance (*p* < 0.05), as compared to cisplatin-treated rats. 

### 3.3. Serum Chemistry

#### Effect of Rosinidin on BUN, Cholesterol, and Phospholipid Levels against Cisplatin-Induced Nephrotoxicity in Rats

**Figure 2:**
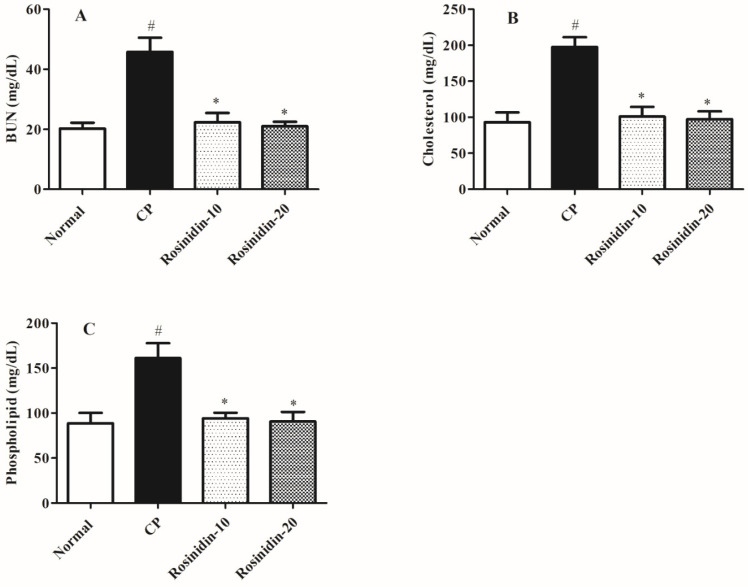
Effect of rosinidin on serum chemistry against cisplatin-induced nephrotoxicity in rats. (**A**) BUN (**B**) Cholesterol (**C**) Phospholipid; Data were presented as mean ± SEM (*n* = 6). # Indicate significant alteration for normal vs. control, * indicated modulations observed in rosinidin vs. cisplatin. *p* value less than 0.05 were considered statistically significant. The analysis was carried out by employing the Kruskal–Wallis test.

Figure 2 revealed the effect of rosinidin on the assessment of several serum chemical profiles in cisplatin-induced nephrotoxicity in rats. In the present study, we observed that in comparison to normal-treated rats, cisplatin-treated rats had substantially elevated levels of several serum chemistry parameters such as BUN, cholesterol, and phospholipid levels (*p* < 0.05). Similarly, in another set of analyses, a high dose of rosinidin (20 mg/kg) notably altered the elevated levels of BUN, cholesterol, and phospholipid levels (*p* < 0.05) as compared with the cisplatin-induced rat group. Besides that, the Kruskal–Wallis test revealed that low-dose rosinidin (10 mg/kg) demonstrated remarkable alterations in the serum chemistry parameters such as BUN (*p* < 0.05), cholesterol (*p* < 0.05), and phospholipid levels (*p* < 0.05) as compared to the cisplatin-induced rat group.

### 3.4. Enzymatic and Non-Enzymatic Parameters

#### Effect of Rosinidin on Levels of Enzymatic and Non-Enzymatic Parameters against Cisplatin-Induced Nephrotoxicity in Rats

**Figure 3:**
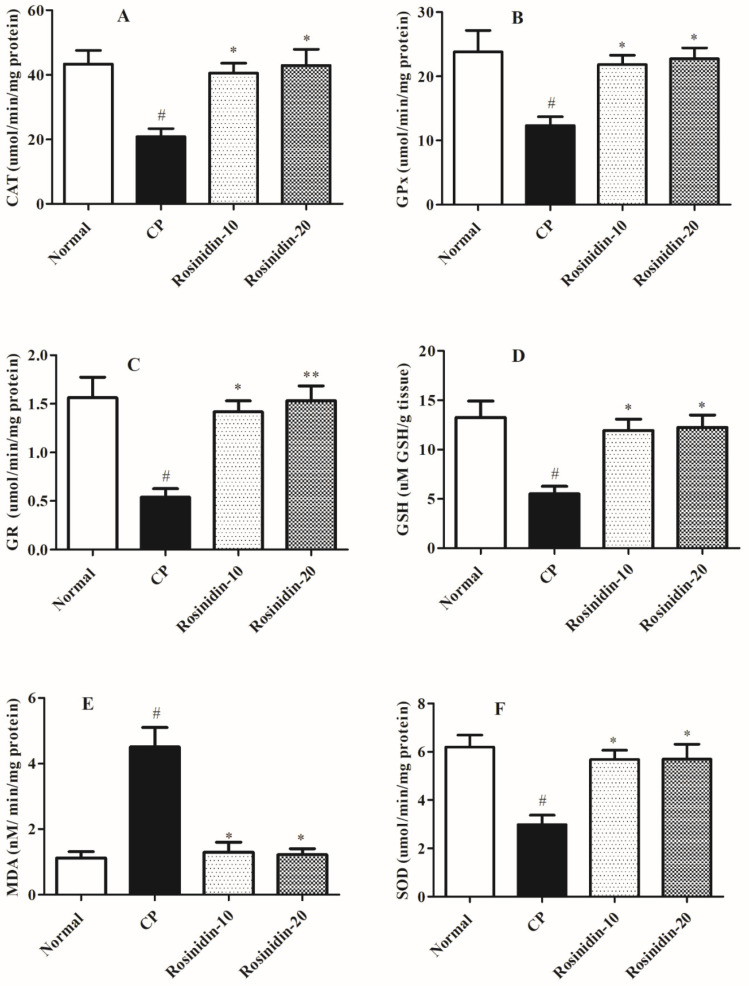
Effect of rosinidin on levels of enzymatic and non-enzymatic parameters against cis-platin-induced nephrotoxicity in rats. (**A**) CAT (**B**) GPx (**C**) GR (**D**) GSH (**E**) MDA (**F**) SOD; Data were presented as mean ± SEM (*n* = 6). # Indicate significant alteration for normal vs. control; ** *p* < 0.01, * *p* < 0.05 indicated modulations observed in rosinidin vs. cisplatin. *p* value less than 0.05 were considered statistically significant. The analysis was carried out by employing the Kruskal–Wallis test.

Figure 3 shows the impact of rosinidin on levels of enzymatic and non-enzymatic parameters in rats against cisplatin-induced nephrotoxicity. In the current analysis, the cisplatin-induced rats showed significant alterations in several enzymatic activities as well as levels of several non-enzymatic biomarkers of oxidative stress which is linked with the nephrotoxic activities in the kidney tissues.

When compared to normal-treated rats, cisplatin-induced rats exhibited a substantial (*p* < 0.05) decline in the enzymatic activities such as CAT, GSH, GR, GPx as well as non-enzymatic biomarker level of SOD in the kidney tissue which is considered a key biomarker for ROS. In another set of experiments, cisplatin-induced rats exhibited considerably elevated levels of MDA as an important oxidative biomarker in kidney tissue (*p* < 0.05). In addition, the Kruskal–Wallis test discovered that a high dose of rosinidin (20 mg/kg) resulted in a significantly restores the activities of several enzymatic parameters which is inhibited in the cisplatin-treated group which CAT (*p* < 0.05), GSH (*p* < 0.05), GR (*p* < 0.01), GPx (*p* < 0.05), and non-enzymatic SOD level (*p* < 0.05). Similarly, a low dose of rosinidin significantly restores the elevated levels of enzymatic and non-enzymatic parameters in cisplatin-induced rats such as CAT (*p* < 0.05), GSH (*p* < 0.05), GR (*p* < 0.05), GPx (*p* < 0.05), and SOD (*p* < 0.05) as well as a significant decline in the levels of oxidative stress biomarkers MDA (*p* < 0.01), which indicate significant alterations associated with chemical-induced nephrotoxicity in the experimental animal models. 

## 4. Discussion

The previous line of research demonstrated the effectiveness of cisplatin as an important chemotherapeutic agent despite its reported adverse events. However, earlier reports also revealed that cisplatin is known to cause acute kidney injury in the majority of patients [41,42]. The cellular mechanism in the pathogenesis of kidney injury involves the generation of reactive oxygen species (ROS) via inhibition of the mitochondrial respiratory complex specifically in cells of renal tubules, which resulted in renal organ and tissue injury [43]. Additionally, clinical hypotheses are made which state that the occurrence of inflammatory and oxidative stress is linked with structural and functional irregularities if any are observed with renal tissues. Previously conducted research studies suggested that several antioxidants have been clinically proven to be effective in the prevention of cisplatin-induced toxicities [32]. These antioxidant agents mainly include vitamin E and vitamin C [32,44,45], several phytoconstituents [46], selenium [47], melatonin [48], along with some nitric oxide modulators that interfere with the metabolic pathways of cisplatin [49,50,51]. The present investigation considered possible nephroprotective efficacy against cisplatin-induced nephrotoxicity in rats. Acute toxicity tests for rosinidin were performed in the assessment to determine any toxicities associated with rosinidin. We observed that rosinidin at dosage of 10 and 20 mg/kg was determined to be safe in rats, with no observed clinical symptoms or conditions.

Previous research has emphasized the neurotoxic potential of the oxidative stress inducer via mitochondrial toxin cisplatin. In the present investigation, we studied cisplatin-induced nephrotoxicity in the experimental animal model. Several preclinical investigations suggested that the administration of several nephrotoxic substances (e.g., cisplatin) in animals extensively altered the body weight of rodents [52]. Similarly, in our study, we found that the cisplatin-induced control-treated animals had a substantial decline in mean body weight when counted at the end of the study. Interestingly, a high dose of rosinidin remarkably increases the mean body weight. 

In the present study, we performed several biochemical parameters to explore the alterations that are linked with cisplatin-induced experimental models of nephrotoxicity. The previous investigation suggested that several biomarkers are generated in the pathology of chemical-induced nephrotoxicity [45]. Additionally, these biochemical markers have primarily highlighted the significance of the estimation of non-protein-nitrogenous components, blood chemistry profiling with activity, and levels of important enzymatic and non-enzymatic biomarkers. Earlier studies explained the involvement of these oxidative parameters in the pathogenesis of nephrotoxicity, with the main focus on GSH, GPx, GR, CAT, SOD, and MDA in the kidney tissues. Furthermore, earlier data also showed that in cisplatin-induced nephrotoxicity there is a substantial decline in the activity of CAT, GSH, GR, GPx, and SOD levels in the kidney tissues. However, remarkably elevated levels of MDA were found while assessing the kidney profiles. In our investigation, we found that the cisplatin-induced rat group exhibited significant alterations in the activity of CAT, GSH, GR, GPx, and SOD in the kidney homogenate along with elevated levels of MDA. Post-treatment with rosinidin for 25 days significantly restored the whole activity as well levels of biomarkers, which is indicative of the nephroprotective efficacy of rosinidin in the cisplatin-induced nephrotoxicity in the rats.

Similarly, previous data also postulated the altered levels of non-protein-nitrogenous components such as serum urea, serum uric acid, serum creatinine, and creatinine clearance in the blood. Furthermore, levels of urine creatinine, urine urea, urine uric acid, and urine volume from collected urine samples in experimental animal models were also shown to be altered [53]. In the present investigation, we considered the levels of non-protein-nitrogenous components in the kidney homogenate. The injection of cisplatin intensely altered the levels of the above-mentioned non-protein components in the 25-day investigational protocol which elaborates the toxic potential of cisplatin against normal physiological functions. In contrast, rosinidin (10 and 20 mg/kg) treatment pointedly recovers the levels of all above-mentioned biological components in rats.

There is extensively distributed evidence suggestive of the significance of blood chemistry in a clinical manifestation of chemical-induced nephrotoxicity [32]. Earlier data also demonstrated that alterations in normal kidney functions are associated with significant changes in the levels of several blood biomarkers of nephrotoxicity, namely BUN, serum cholesterol, and phospholipid levels s [54]. In our study, we revealed that the injection of cisplatin considerably affected the serum levels of BUN, serum cholesterol, and phospholipid. Similarly, in our study, we observed that the administration of several doses of rosinidin in rats during a 25-day protocol significantly improves the blood chemistry profiles, which indicated that rosinidin has significant nephroprotective efficacy.

## 5. Conclusions

The present study evaluated, for the first time, data claiming rosinidin as a potent nephroprotective efficacy, the ability to improve several biochemical markers of oxidative stress parameters with abnormal enzymatic and non-enzymatic components, and altered blood chemistry profiles in the cisplatin-induced nephrotoxicity the experimental animal models. Moreover, the ameliorative efficacy of rosinidin in non-protein-nitrogenous components showed that rosinidin has nephroprotective properties. 

This might lead to the advance of cost-effective phytomedicine possibilities for the treatment of drug-induced complications with great emphasis on nephrotoxicity. The above evidence demonstrated that rosinidin has the potential to be a beneficial natural component in the treatment of drug-associated nephrotoxic complications.

## Data Availability

Not applicable.
